# The Utilisation, Knowledge and Opinions Regarding Static Computer‐Assisted Implant Surgery (s‐CAIS) Among Australian and New Zealand Dental Practitioners: A Survey

**DOI:** 10.1155/ijod/6753844

**Published:** 2026-06-29

**Authors:** Seung Hyun Kim, Toby Hughes, Alan Broughton, Andrew Cheng, Sushil Kaur

**Affiliations:** ^1^ School of Dentistry, College of Health, Adelaide University, Adelaide, South Australia, Australia, adelaide.edu.au

**Keywords:** Australia, dental implant, fully-guided surgery, general dental practitioner, guided implant surgery, New Zealand, specialist dental practitioner

## Abstract

**Background:**

Increasingly, static computer‐assisted implant surgeries (s‐CAISs) are being promoted to dental practitioners with warranties of providing high levels of accuracy and precision in achieving the pre‐planned implant position. However, there is a lack of evidence in the current literature regarding the accuracy of s‐CAIS in certain clinical situations. In light of the paucity of evidence, this study aimed to identify the utilisation, knowledge and opinions regarding s‐CAIS among Australian and New Zealand practitioners involved in clinical practice.

**Methods:**

Anonymous online surveys were distributed to various dental associations, tertiary organisations and implant companies. R (R Core Team, 2021) and RStudio (RStudio Team, 2020) software were utilised for logistic regression analyses.

**Results:**

A total of 304 responses were received. Of the valid responses (*n* = 267), excluding the 59 participants who had not surgically placed dental implants, 81% of clinicians had utilised s‐CAIS at least once. Variations in adopting s‐CAIS were observed across practitioners of different specialties, years in clinical practice and the number of implants surgically placed. Generally, general dental practitioners (GDPs) and clinicians with less clinical experience tended to view the advantages of s‐CAIS more favourably.

**Conclusion:**

This survey identifies a need for continuing education regarding implant treatment planning and the factors that may influence the accuracy of s‐CAIS, for clinicians who currently utilise or intend to utilise the technology in the future.

## 1. Introduction

The clinical success and predictability of osseointegrated dental implants have led to their wide acceptance in oral rehabilitation [1, 2]. From single implant crowns and multiple‐unit fixed dental prostheses (FDPs) to implant overdentures, dental implants offer alternative and complementary solutions compared to conventional fixed or removable prostheses to replace missing teeth in partially dentate and fully edentulous ridges.

Despite the clinical successes afforded by the addition of osseointegration to the available treatment armamentarium, the surgical placement of the implant in the pre‐planned position is rarely straightforward. Limited visual and tactile access to the operative field, patient movement during osteotomy, and constraints in operators’ ability to accurately translate planned implant placement to the intra‐operative setting can pose significant challenges for free‐handed (FH) surgery.

The fixture/s must, nevertheless, be inserted in the ideal three‐dimensional (3D) position to construct a favourable prosthesis that will fulfil the patient’s functional and aesthetic demands. Poorly placed implants may increase the risk of complications, including marginal bone resorption [3]. Moreover, the ideal implant position will allow for a prosthetic design compatible with long‐term maintenance and facilitate accessible oral hygiene practices [4]. Finally, appropriate surgical and restorative planning and their execution will improve the likelihood of achieving a screw‐retained prosthetic design, thereby preventing potential complications associated with cemented restorations [5].

The available evidence suggests that implant placement in a prosthetically driven ideal 3D position is a key constituent for long‐term success. Judicious pre‐surgical implant and prosthetic planning are paramount criteria for treatment success. With advancements in technology, clinicians are now able to plan the intended implant position, which can be transferred intraorally by incorporating dynamic or static guidance systems following digital implant planning.

Static‐guided surgery involves the preoperative construction and intraoperative utilisation of a rigid surgical stent [6]. The accuracy of static computer‐assisted implant surgery (s‐CAIS) has been well documented [7, 8]. However, the workflow for s‐CAIS may vary considerably between clinicians, from the pre‐operative and intra‐operative to the post‐operative stages (impression acquisition for the construction of the implant prosthesis) of treatment. Furthermore, various patient‐related factors, including the degree of mouth opening, the length of the edentulous ridge and the number and location of teeth available to retain the surgical guide, have been documented to potentially affect the overall accuracy of transferring the pre‐planned position to the surgical site [9–11]. Finally, few publications have been reported regarding the utilisation of s‐CAIS in fully edentulous patients as opposed to partially dentate.

In light of the discrepancy between the current literature and the utilisation of s‐CAIS in clinical practice, this survey aimed to determine the following:

1. The prevalence of s‐CAIS among Australian and New Zealand dental practitioners.
2. The adoption of s‐CAIS usage among dental practitioners, such as the methods of initial data acquisition, their extent of involvement in planning the case digitally, characteristics of patients selected for s‐CAIS and complications (surgical and/or prosthetic) encountered.
3. Practitioners’ knowledge and opinions toward s‐CAIS regarding its accuracy, reliability and adequacy in clinical implant practice.
4. The potential influence of specialisation, the number of years in clinical practice and the number of implants surgically placed on the outcome.

## 2. Materials and Methods

### 2.1. Survey Participants

The inclusion criteria for this survey were general dental practitioners (GDPs) and specialist dental practitioners (SDPs) of all disciplines currently registered and practising in Australia or New Zealand. Post‐graduate students could also participate in the survey if they maintained their recency in clinical practice outside the university. Participants were requested not to participate if they were under 18 or those maintained an undergraduate student status.

### 2.2. Survey Design

An initial pilot survey was distributed to 23 dental practitioners for feedback. The final questionnaire was created online via SurveyMonkey (San Mateo, CA, USA), which consisted of a combination of multiple‐choice and Likert scale questions, with options for providing additional comments where appropriate. Appropriate skip logic questions were utilised for the survey to remain relevant for all respondents who fulfilled the inclusion criteria. The final survey consisted of 31 questions in three sections. The first section enquired about the participant demographics including practice location, area of specialty, years of experience and the total number of dental implants surgically placed. The second section explored how s‐CAIS was adopted into clinical practice including case selection, case planning, surgical protocol and the main method of impression acquisition. The third section enquired about the participants’ knowledge and opinions of s‐CAIS, regardless of surgical experience. Upon completion of the survey, respondents were able to enter into an optional draw for gift vouchers as a token of appreciation.

### 2.3. Survey Distribution

Various dental organisations were approached for voluntary survey distribution (Table 1).

**Table 1:** List and abbreviations of dental organisations involved in survey distribution.

Dental associations
Australian Dental Association Federal (ADA) Australian Dental Association New South Wales (ADANSW) Australian Dental Association Victoria (ADAVB) Australian Dental Association Queensland (ADAQ) Australian Dental Association Western Australia (ADAWA) Australian Dental Association South Australia (ADASA) Australian Dental Association Northern Territory (ADANT) Australian Dental Association Tasmania (ADATB) New Zealand Dental Association (NZDA) Australian and New Zealand Academy of Periodontists (ANZAP) Australian and New Zealand Association of Oral and Maxillofacial Surgeons (ANZAOMS) Australian Prosthodontic Society (APS) Australian Society of Periodontology (ASP) Academy of Australian and New Zealand Prosthodontists (AANZP) International Team for Implantology (ITI)
Tertiary organisations
The University of Sydney – Graduate Diploma in Clinical Dentistry (Oral Implants): 2018, 2020 and 2022 cohorts The University of Melbourne – Graduate Diploma in Clinical Dentistry (Implants): 2022 cohort
Implant companies
Nobel Biocare MIS Dentium

The predominant method of survey distribution was via inclusion in e‐newsletters or by purpose‐specific individual emails. For the Australian Dental Association Queensland (ADAQ) branch, the survey link was presented on an online social media platform.

### 2.4. Statistical Analysis

Logistic regression analyses were implemented to compare the results by specialty, the number of years in clinical practice and the number of implants surgically placed. R [12] and RStudio [13] softwares with packages Tidyverse [14], multcomp [15] and nnet [16] were employed for the regression analyses. The number of years practised and the number of implants placed were assumed to be co‐linear. The outcomes of the regression analyses were reported as odds ratios (ORs) and the range of confidence intervals (CIs) at 95%.

### 2.5. Ethics

Ethics approval for this research project was granted by the Adelaide University (H‐2021‐213).

## 3. Results

### 3.1. Demographics

**Figure 1:**
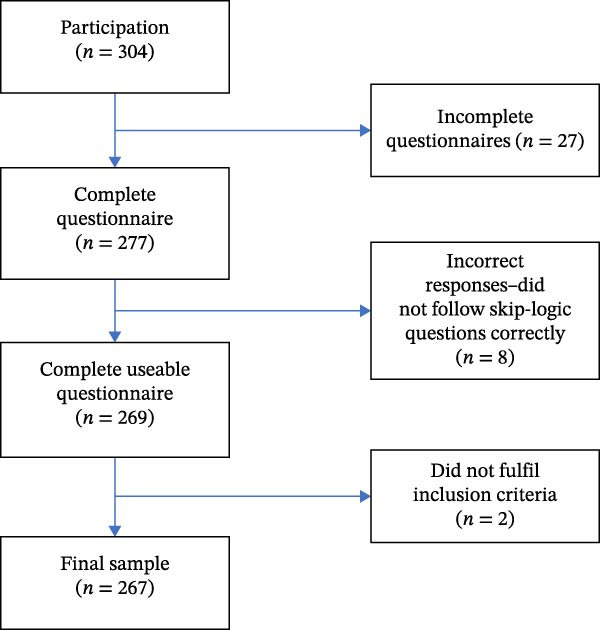
Determination of the final sample size.

A total of 22,281 practitioners were approached for participation in this survey. Given that 304 responses were received in total, this equated to a response rate of 1.36%. With the exclusion of incomplete questionnaires, responses that incorrectly followed the skip‐logic questions, and responses from participants who did not fulfil the inclusion criteria, the final sample totalled 267 participants (Figure 1).

Table 2 summarises the respondents by geographical distribution, occupation, the number of years in practice and the number of implants surgically placed.

**Table 2:** Surveyed practitioner characteristics by geographical distribution, occupation, the number of years in practice and the number of implants surgically placed (*N* = 267).

Geographical distribution	Number and percentage of participants
ACT	3 (1.1%)
NSW	70 (26.2%)
NT	10 (3.7%)
Qld	25 (9.4%)
SA	85 (31.8%)
Tas	3 (1.1%)
Vic	41 (15.4%)
WA	14 (5.2%)
NZ	16 (6.1%)
Occupation	
GDP	199 (74.5%)
SDP	68 (25.5%)
Number of years in practice	
0–5 years	39 (14.6%)
6–10 years	55 (20.6%)
11–20 years	80 (30.0%)
>20 years	93 (34.8%)
Number of implants surgically placed	
None	59 (22.1%)
1–50	59 (22.1%)
50–200	44 (16.5%)
200–1000	52 (19.5%)
1000–2500	28 (10.5%)
>2500	25 (9.4%)
Total response	267

### 3.2. Adoption Rate of s‐CAIS

Of all survey participants, 77.9% (*n* = 208) had surgically placed at least one dental implant.

In this study, among the 208 clinicians with surgical implant experience, 81.3% (*n* = 169) had utilised s‐CAIS at least once. These findings were similar between GDPs (79.8%) and SDPs (85.2%). Logistic regression analysis revealed no influence of specialty, the number of years in practice or the number of implants placed on having ever utilised s‐CAIS.

Of the 39 practitioners with surgical implant experience who had not utilised s‐CAIS, 69.2% (*n* = 27) responded that they would consider using s‐CAIS for future implant surgeries.

### 3.3. Frequency of s‐CAIS Utilisation

**Figure 2:**
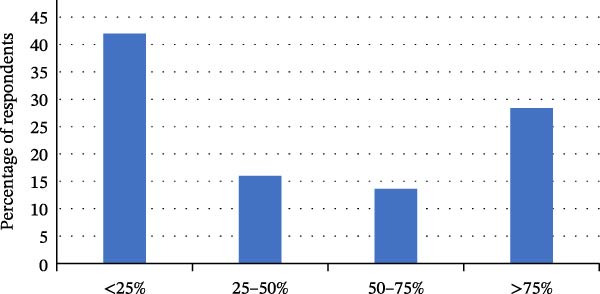
Percentage of implant cases completed by s‐CAIS.

Among practitioners with s‐CAIS experience, a broad range of frequencies was observed regarding how often they had adopted the technology for their implant cases (Figure 2).

### 3.4. Case Selection

More than four‐fifths of respondents (82.8%) utilised s‐CAIS for both anterior (incisor and canine) and posterior (pre‐molar and molar) edentulous sites, while 12.4% indicated they had utilised s‐CAIS for posterior sites only, and 4.7% responded that they had adopted s‐CAIS for anterior sites only.

55% of respondents utilised s‐CAIS for both partially dentate and fully edentulous patients, while 43.8% utilised s‐CAIS for partially dentate patients only and 1.2% utilised the technology for fully edentulous patients only.

Three‐quarters (75.1%) of clinicians utilised s‐CAIS for single‐unit (i.e., Tooth 13) and multiple‐unit (i.e., Teeth 13 and 14) cases. 13.6% of clinicians used s‐CAIS for multiple‐unit cases only, while 11.2% utilised s‐CAIS solely for single‐unit cases.

More than three‐quarters (78.7%) of clinicians utilised s‐CAIS for both bounded and free end saddle cases, while 15.4% utilised s‐CAIS for bounded saddle cases only and 5.9% utilised s‐CAIS for free end saddle cases only.

Overall, surveyed practitioners with s‐CAIS experience appeared to have adopted the technology widely in various clinical scenarios.

### 3.5. Case Planning

**Figure 3:**
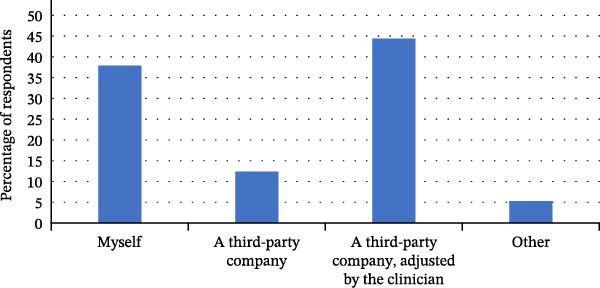
Involvement in digitally planning the implant position for the majority of s‐CAIS cases.

Variable responses were observed regarding clinicians’ involvement in digital implant position planning (Figure 3). Overall, more than half (56.8%) of the s‐CAIS cases were found to be fully planned or influenced by a third‐party company or a dental laboratory.

### 3.6. Implementation of s‐CAIS: Level of Guidance

**Figure 4:**
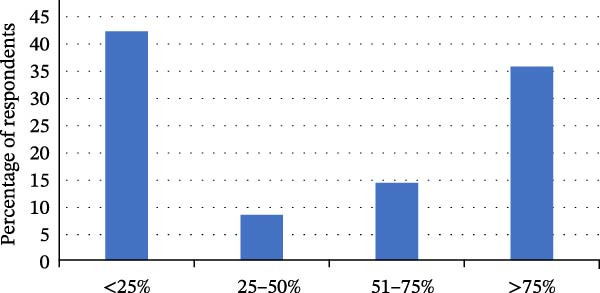
Percentage of s‐CAIS completed as FG surgery.

For cases completed by s‐CAIS, clinicians were queried regarding the percentage of cases completed fully guided (FG; drilling sequence and implant placement through the surgical guide), as summarised in Figure 4.

### 3.7. Implant Impression

In this study, 47.9% of respondents chose conventional dental impression materials (i.e., polyvinyl siloxane) as their main method of impression acquisition for the final implant prosthesis. 37.3% of respondents used an intra‐oral scanner (IOS), while 14.8% responded as ‘unsure’ as they were not involved in the final prosthesis construction.

### 3.8. Outcomes of s‐CAIS

Table 3 demonstrates that among practitioners with s‐CAIS experience, 95.7% of practitioners mentioned they had encountered unforeseen complications intra‐operatively during usage of s‐CAIS, of varying frequencies. In addition, 85.2% of practitioners have had to modify the final implant position from the pre‐planned position. Despite the above experiences, 95.8% of practitioners replied that they were able to restore the implants according to the original plan either ‘mostly’ or ‘always’. In addition, 97.1% of s‐CAIS users responded either ‘mostly’ or ‘always’ to fulfilling patient expectations.

**Table 3:** Participant responses regarding their experiences with s‐CAIS.

Survey questionnaires	Always (%)	Mostly (%)	Sometimes (%)	Rarely (%)	Never (%)
*N* = 169					
Did you run into unforeseen complications intra‐operatively?	1.2	8.9	43.2	41.4	5.3
Did you have to modify the final implant position from the planned position?	1.8	4.1	43.8	35.5	14.8
Were the implants able to be restored according to the original plan?	41.4	54.4	3.6	0.6	0.0
Did the outcome (surgical and restorative) meet the patient’s expectations?	39.1	58.0	1.8	1.2	0.0

### 3.9. Knowledge and Opinions of s‐CAIS

All practitioners who fulfilled the inclusion criteria were requested to comment on their knowledge and opinion of s‐CAIS, regardless of whether they had surgically placed dental implants or previously utilised the technology (Table 4).

**Table 4:** Respondent data regarding their knowledge and opinions of s‐CAIS.

Survey questionnaires	Always (%)	Mostly (%)	Sometimes (%)	Rarely (%)	Never (%)	Unsure (%)
*N* = 267						
a) Helps to increase case acceptance	8.2	28.8	29.2	11.6	4.5	17.6
b) Helps in accurate implant positioning	29.2	55.1	10.9	1.1	0.4	3.4
c) Reduces the number of radiographs (i.e., periapical films) taken during surgery	16.5	28.1	27.0	10.1	6.7	11.6
d) Helps to reduce intraoperative complications	10.9	49.8	19.5	8.2	1.9	9.7
e) Helps to reduce surgery time	22.1	42.7	23.2	4.5	1.5	6.0
f) Helps to reduce patient discomfort (intra‐ and post‐operatively)	10.9	27.7	25.5	12.0	6.0	18.0
g) Helps to improve overall patient experience	16.1	36.7	26.2	6.4	2.2	12.4
h) Helps to provide optimal aesthetic outcomes	13.1	46.1	22.8	4.5	1.5	12.0
i) Is surgically easier than non‐guided implant surgeries	19.5	36.0	21.7	8.2	5.2	9.4
j) Requires less surgical implant experience than non‐guided surgery	15.4	24.3	17.6	10.9	18.7	13.1

## 4. Discussion

### 4.1. s‐CAIS Utilisation

In this study, four out of five clinicians (81.3%) who had surgically placed dental implants had utilised s‐CAIS. This figure was significantly higher than two other publications, which reported on the prevalence of s‐CAIS utilisation in Saudi Arabia [17] and central India [18], ranging from 40% to 46%. However, both investigations must be interpreted with caution given the low number of survey respondents and the possibility of selection bias.

The comparison of the outcomes by clinician groups revealed that GDPs were significantly more likely (OR = 4.8, 95% CI [2.19, 10.82]) to have utilised s‐CAIS in a larger percentage of their implant cases than SDPs. This observation may indicate a higher tendency for GDPs to embrace newer technology in daily clinical practice compared to SDPs. In addition, frequent s‐CAIS utilisation by GDPs may be associated with their positive attitudes, knowledge and or opinions toward the technology. Furthermore, clinicians who had placed 1000–2500 (OR = 7.4, 95% CI [1.33, 50]) or >2500 implants (OR = 16.3, 95% CI [2.22, 100]) were significantly more likely to have utilised s‐CAIS in a smaller percentage of their implant cases compared to clinicians who had placed 1–50 implants. These outcomes may be partly associated with the recency of the technology, whereby more experienced clinicians had already performed implant surgery conventionally for a longer period, as opposed to less experienced clinicians who were exposed to s‐CAIS for a greater proportion of their careers. Alternatively, these observations may suggest the greater tendency for less experienced clinicians to rely on s‐CAIS for their implant surgeries given the comparative lack of surgical experience. Overall, s‐CAIS was utilised more often (among implant cases completed) by GDPs than SDPs and by practitioners who surgically placed fewer implants. To the author’s knowledge, no other publications have reported on the frequency of s‐CAIS utilisation or compared the outcomes by specialty, years in practice and the number of implants placed.

### 4.2. Case Selection

Survey participants were queried regarding their utilisation of s‐CAIS for partially dentate, fully edentulous patients or both clinical scenarios. Despite the comparative scarcity in the current literature supporting the high accuracy of s‐CAIS for fully edentulous patients, more than half (53.8%) of clinicians indicated that they had utilised s‐CAIS in these patients. These observations may be attributable to clinicians’ previous experiences or beliefs that support s‐CAIS in fully edentulous patients as opposed to FH surgery. It is also conceivable that with rapid advancements in technology, s‐CAIS for fully edentulous patients may have attained higher levels of accuracy compared to outcomes published in the currently available literature and that outcomes for these newer methodologies have yet to be published.

### 4.3. Case Planning

Practising implant dentistry not only encompasses sound intra‐operative skills but also includes conducting judicious pre‐surgical implant planning. This includes a full assessment of the patient’s medical history, periodontal and prosthodontic status to determine whether dental implants are suitable or preferable for the patients’ oral rehabilitation. During the implant site assessment, the condition of adjacent teeth, proximity to major anatomical structures, the degree of alveolar bone cortication and the evaluation of any soft and hard tissue deficiencies will enable the appropriate selection of implant placement timing, location, shape, width and length.

In a prospective study, Raabe et al. [19] evaluated inter‐ and intra‐operator variability in digital single‐tooth implant positioning among practitioners with varying clinical experiences. Six novice and six expert clinicians in oral surgery and prosthodontics virtually planned the implant positions of 15 patients at two separate time points. Although substantial inter‐ and intra‐individual variability was observed, significantly lower deviations were recorded in the expert group, highlighting the potential influence of experience in digital implant position planning.

In the present study, more than half of the s‐CAIS cases were found to be fully planned or influenced by a third‐party company. Logistic regression analyses revealed that practitioners with 11–20 years (OR = 14.3, 95% CI [2.73, 75.3]) or >20 years (OR = 7.3, 95% CI [1.41, 37.8]) of clinical experience were significantly more likely than clinicians with 1–5 years of experience to utilise a third party and adjust the implant position themselves where necessary, as opposed to delegating the implant position planning entirely to a third party. Second, a comparison of outcomes by the number of implants placed revealed clinicians who had placed 200–1000 implants were more likely (OR = 6.1, 95% CI [1.13, 32.81]) than those who placed 1–50 implants to utilise a third party for implant position planning (with necessary adjustments) rather than relying solely on a third party. Moreover, clinicians who placed 200–1000 (OR = 6.1, 95% CI [1.13, 32.81]) or 1000–2500 implants (OR = 16.1, 95% CI [1.55, 167.22]) were more likely to plan implant positions independently rather than relying on a third party. Collectively, these outcomes suggest that employing a third party for digital implant site planning was not uncommon among practitioners of all experiences, although with more clinical experience, practitioners generally tended to be less reliant on third parties. Such observations raise concerns given that the high accuracy of s‐CAIS is predicated on meticulous and careful pre‐surgical planning by a well‐trained dental professional [19, 20]. Furthermore, third parties may lack awareness of patient‐specific contextual factors, including the degree of mouth opening and the flexibility of oral soft tissues for retraction, altering utilisation of the surgical stent. Consequently, for inexperienced clinicians, over‐reliance on s‐CAIS without freehanded surgery proficiency may diminish the ability to anticipate and manage unforeseen intraoperative challenges.

### 4.4. Level of Surgical Guidance

Current evidence supports the superior accuracy of FG s‐CAIS compared with partially guided (PG) s‐CAIS and freehand surgery in partially dentate patients [8, 21, 22]. In this study, GDPs were significantly more likely than SDPs (OR = 4.4, 95% CI [1.99, 9.79]) to use FG approaches in a greater proportion of s‐CAIS cases. Although not statistically significant, less experienced clinicians also tended to favour FG over PG techniques. This pattern may reflect greater reliance on surgical guides among GDPs and less experienced clinicians, whereas SDPs and more experienced practitioners may prefer intraoperative control based on their surgical expertise.

### 4.5. Implant Impression (for Final Prosthesis)

In this study, conventional impressions remained the most used method for final implant prostheses following s‐CAIS. However, after excluding clinicians not involved in impression acquisition, 43.7% reported intra‐oral scanning (IOS) as their primary method, with IOS use exceeding 30% across all clinician subgroups. These outcomes are comparable to those reported by Revilla‐Leon et al. [23], who found that 53% of 369 practitioners surveyed in the United States indicated they had adopted IOS into general clinical practice. Overall, this study demonstrates the strong uptake of digital technologies for implant impression acquisition across clinicians in Australia and New Zealand, regardless of clinical or surgical implant experience. With the advancement of technology and reduction of IOS utilisation costs, fully digital workflows are expected to become increasingly prevalent.

Although not statistically significant, clinicians who had placed a greater number of implants were less likely to be involved in impression acquisition for the final prosthesis. This may reflect a tendency among more experienced practitioners to focus on surgical treatment and delegate prosthetic procedures or to place implants for referred cases that are subsequently returned to the referring clinician for impression acquisition.

### 4.6. Knowledge and Opinions of s‐CAIS

In this study, all participants who met the inclusion criteria (including those with no surgical implant experience) were asked to comment on their knowledge and opinion of s‐CAIS.

Although influenced by multiple pre‐ and intra‐surgical factors, the precision and accuracy of s‐CAIS in achieving planned implant positioning have been well documented in both model‐based and in vivo studies [21, 22]. In the present survey, participants’ responses were in accordance with the current evidence, with more than four‐fifths (84.3%) indicating that s‐CAIS ‘mostly’ or ‘always’ aids accurate implant positioning.

Some studies [24, 25] reported that flapless s‐CAIS may help to reduce post‐operative discomfort compared to conventional open‐flap surgery. However, recent systematic reviews [26, 27] have not demonstrated superiority of s‐CAIS over non‐guided surgery regarding post‐operative discomfort. Interpretation of the existing evidence is limited by heterogeneity in study design and outcome measures, including analgesic consumption, visual analogue scales (VASs), patient‐reported questionnaires and assessment of post‐surgical swelling. In addition, post‐operative discomfort is likely to be more influenced by flap elevation as opposed to implant placement with or without a surgical guide. In this study, no clear correlation in opinions was observed regarding whether s‐CAIS aids in reducing post‐operative patient discomfort.

In general, the comparison of outcomes between various clinician groups by ordinal logistic regression analyses revealed that overall, GDPs tended to view the advantages of s‐CAIS more favourably. GDPs were more likely to believe that s‐CAIS helped increase implant case acceptance (OR = 2.7, CI 95% [1.34, 5.61]), reduce surgery time (OR = 2.0, CI 95% [1.09, 3.65]) and improve overall patient experience (OR = 2.1, CI 95% [1.07, 3.97]) compared to SDPs. Furthermore, practitioners who had placed 1–50 implants were more likely to believe that s‐CAIS helps to reduce surgery time (OR = 7.2, CI 95% [1.40, 33.33]) and improve overall patient experience (OR = 6.5, CI 95% [1.25, 33.33]) compared to practitioners who placed >2500 implants.

The influence of operator experience on the outcome of s‐CAIS has been published in a few studies. A model‐based study by Rungcharassaeng et al. [20] evaluated the effects of operator experience on the accuracy of s‐CAIS. While no significant differences were observed in angular or linear deviations, the degree of vertical deviation of the implant by inexperienced clinicians was approximately double when compared to that by experienced clinicians, although not statistically significant. A randomised controlled pilot study by Casetta and Bellardini [28] compared the accuracy of s‐CAIS performed by clinicians with varying surgical experience in fully edentulous patients. No significant differences were reported between groups in coronal, apical, or angular deviations between the planned and actual implant positions. Nevertheless, guide positioning errors attributable to guide placement errors were significantly more frequent among inexperienced clinicians. Based on the outcomes, the authors dismissed the belief that s‐CAIS requires less surgical experience.

In the present study, clinicians in the early stages of practice (1–5 years in clinical practice and/or 1–50 implants surgically placed) demonstrated perceptions of certain aspects of s‐CAIS that are not aligned with the available evidence. Practitioners with 1–5 years of clinical experience were more likely than those with 11–20 years (OR = 5.2; 95% CI [1.29, 20.0]) or >20 years of experience (OR = 3.7; 95% CI [1.12, 12.5]) to believe that s‐CAIS is surgically easier than non‐guided surgery. This group was also more likely than clinicians with 11–20 years (OR = 3.7; 95% CI [1.12, 12.5]) or >20 years of experience (OR = 4.1; 95% CI [1.29, 14.28]) to believe that s‐CAIS requires less surgical implant experience than non‐guided surgery. In addition, clinicians who had placed 1–50 implants were more likely to hold this view (OR = 7.2; 95% CI [1.92, 25.0]) compared with those who had placed 200–1000 implants.

While the superior accuracy of s‐CAIS over non‐guided surgeries in partially edentulous cases is well documented [21, 22], various factors may affect the accuracy and, thus, alter the final implant position. These include the degree of mouth opening, the length of the edentulous ridge, maxillary sinus and alveolar ridge morphology, recency of tooth extraction, the number and location of teeth available for retaining the surgical guide, the thickness of the underlying mucosal tissues, the distance from the drill sleeve to the osteotomy site and osteotomy drilling sequences [9, 10, 29–33]. As such, although some complications during s‐CAIS overlap those that may arise during non‐guided surgeries, clinicians must anticipate potential issues unique to s‐CAIS. These include limited access to the implant site and fractured or ill‐fitting surgical guides. This further highlights the need for careful pre‐operative treatment planning, clinical dexterity and maturity to problem‐solve and revert to FH surgery as required. Furthermore, in sites with soft/hard tissue deficiencies such as the anterior aesthetic zone, surgical skills in flapless s‐CAIS alone will be inadequate to sufficiently manage the patient. Finally, given the paucity of literature regarding long‐term functional and aesthetic outcomes of s‐CAIS among clinicians with varying experiences, the notion that s‐CAIS is easier than non‐guided surgeries should be reconsidered.

### 4.7. Limitations of Study

The results of the current investigation need to be interpreted with care, given the limitations of the study. First, a low number of responses were received. Two hundred sixty‐seven responses represent just above 1% of the total number of GDPs and SDPs in Australia and New Zealand in clinical practice, limiting extrapolation of outcomes nationally. Second, a notable variation in response rates was observed across different states and jurisdictions. In this study, SA and NT recorded response rates of 4.11% and 14.2%, respectively. In contrast, the response rates of all other jurisdictions did not exceed 1%. The relative over‐representation of SA and NT may be associated with the methodologies in survey distribution, potentially leading to sampling bias. Thirdly, the sources of participant recruitment included implant‐specific organisations such as the ITI, AOS and various implant companies. The responses from the aforementioned sources may have led to selection bias, where practitioners interested in dental implants may have been more interested in responding to this survey. Finally, for ease of analysis, all specialists were categorised into the ‘SDP’ group, enabling pairwise comparisons of s‐CAIS utilisation between SDPs and GDPs. While it is reasonable to assume that prosthodontists, periodontists, oral surgeons, and oral and maxillofacial surgeons have received formal tertiary education regarding dental implants, this is less likely to be true for specialists of other disciplines. Thus, the comparison of outcomes between GDPs and SDPs needs to be interpreted with some caution.

## 5. Conclusion

Being mindful of the shortcomings, nearly four out of five clinicians with prior surgical implant experience were found to have used s‐CAIS at least once. Variation in s‐CAIS adoption was evident across pre‐operative planning, surgical protocols and impression workflows.

Discrepancies between the evidence base and participant perceptions were also observed, with GDPs and less experienced clinicians generally viewing the advantages of s‐CAIS more favourably.

For clinicians intending to adopt s‐CAIS, further training and/or collaboration with experienced operators are recommended to support independent treatment planning and reduce reliance on third‐party providers. Training should emphasise factors affecting s‐CAIS accuracy across various clinical scenarios. A minimum 2 mm safety margin to adjacent anatomical structures should be maintained, and clinicians must be able to revert to FH surgery where necessary. s‐CAIS should be regarded as an adjunct and not a substitute for sound implant planning and surgical competence required for safe, prosthetically driven implant placement.

## Author Contributions

Seung Hyun Kim wrote the original manuscript draft (lead) and shared responsibility for conceptualising the research (lead), data curation (equal), investigation (lead), project administration (lead) and final review and editing (equal). Toby Hughes developed aspects of the methodology (lead), led the formal analysis (lead) and shared responsibility for data curation (equal), final review and editing (equal) and supervision (equal) of the lead author. Alan Broughton shared responsibility for final review and editing (equal). Andrew Cheng shared responsibility for conceptualising the research (equal). Sushil Kaur shared responsibility for supervision (equal) of the lead author and final review and editing (equal).

## Funding

This study was funded by the School of Dentistry, Adelaide University.

## Disclosure

All authors have made significant contributions to this research.

## Ethics Statement

Ethics approval for this research project was granted by the Adelaide University Human Research Ethics Committee (H‐2021‐213).

## Conflicts of Interest

The authors declare no conflicts of interest.

## Data Availability

The deidentified data that support the findings of this study are available upon request from the corresponding author. The data are not publicly available due to privacy or ethical restrictions.
